# BETting on a Transcriptional Deficit as the Main Cause for Cornelia de Lange Syndrome

**DOI:** 10.3389/fmolb.2021.709232

**Published:** 2021-07-27

**Authors:** Pablo García-Gutiérrez, Mario García-Domínguez

**Affiliations:** Andalusian Centre for Molecular Biology and Regenerative Medicine-CABIMER, CSIC-Universidad de Sevilla-Universidad Pablo de Olavide, Seville, Spain

**Keywords:** CdLS, NIPBL, BRD4, transcription, transcriptomopathy

## Abstract

Cornelia de Lange Syndrome (CdLS) is a human developmental syndrome with complex multisystem phenotypic features. It has been traditionally considered a cohesinopathy together with other phenotypically related diseases because of their association with mutations in subunits of the cohesin complex. Despite some overlap, the clinical manifestations of cohesinopathies vary considerably and, although their precise molecular mechanisms are not well defined yet, the potential pathomechanisms underlying these diverse developmental defects have been theoretically linked to alterations of the cohesin complex function. The cohesin complex plays a critical role in sister chromatid cohesion, but this function is not affected in CdLS. In the last decades, a non-cohesion-related function of this complex on transcriptional regulation has been well established and CdLS pathoetiology has been recently associated to gene expression deregulation. Up to 70% of CdLS cases are linked to mutations in the cohesin-loading factor *NIPBL*, which has been shown to play a prominent function on chromatin architecture and transcriptional regulation. Therefore, it has been suggested that CdLS can be considered a transcriptomopathy. Actually, CdLS-like phenotypes have been associated to mutations in chromatin-associated proteins, as KMT2A, AFF4, EP300, TAF6, SETD5, SMARCB1, MAU2, ZMYND11, MED13L, PHIP, ARID1B, NAA10, BRD4 or ANKRD11, most of which have no known direct association with cohesin. In the case of BRD4, a critical highly investigated transcriptional coregulator, an interaction with NIPBL has been recently revealed, providing evidence on their cooperation in transcriptional regulation of developmentally important genes. This new finding reinforces the notion of an altered gene expression program during development as the major etiological basis for CdLS. In this review, we intend to integrate the recent available evidence on the molecular mechanisms underlying the clinical manifestations of CdLS, highlighting data that favors a transcription-centered framework, which support the idea that CdLS could be conceptualized as a transcriptomopathy.

## Introduction

Cornelia de Lange syndrome (CdLS: OMIM 122470, 300590, 300882, 610759, and 614701) is a rare developmental disease with multisystemic effects and variable physical, cognitive and behavioral characteristics. It was first described in 1933 by the Dutch pediatrician Cornelia Catharina de Lange (de Lange, 1933) and its prevalence has been estimated to be between 1/10,000 to 1/30,000 live births (Kline et al., 2007) but, as a consequence of its heterogeneity and the appearance of mild cases and non-classical phenotypes, the incidence and prevalence are probably underestimated. The typical clinical manifestations include restricted growth with prenatal onset, intellectual disability (from mild to profound), craniofacial abnormalities (arched eyebrows with synophrys, long and smooth philtrum, thin upper vermilion), hirsutism and upper limb defects (Cascella and Muzio, 2021). However, this so-called “classic” phenotype is not equally present in all cases and the specific features and the severity of the disorder can vary widely, which leads to the overall characterization of CdLS phenotype as a spectrum (Kline et al., 2018). This CdLS spectrum takes into consideration other clinical findings as gastrointestinal malformations or cardiovascular anomalies and it includes both the classic CdLS phenotype as well as similar non-classic phenotypes, which together with the overlap of some clinical features with other genetic syndromes, contributes to the complexity of the clinical diagnosis of CdLS (Kline et al., 2018).

**FIGURE 1:**
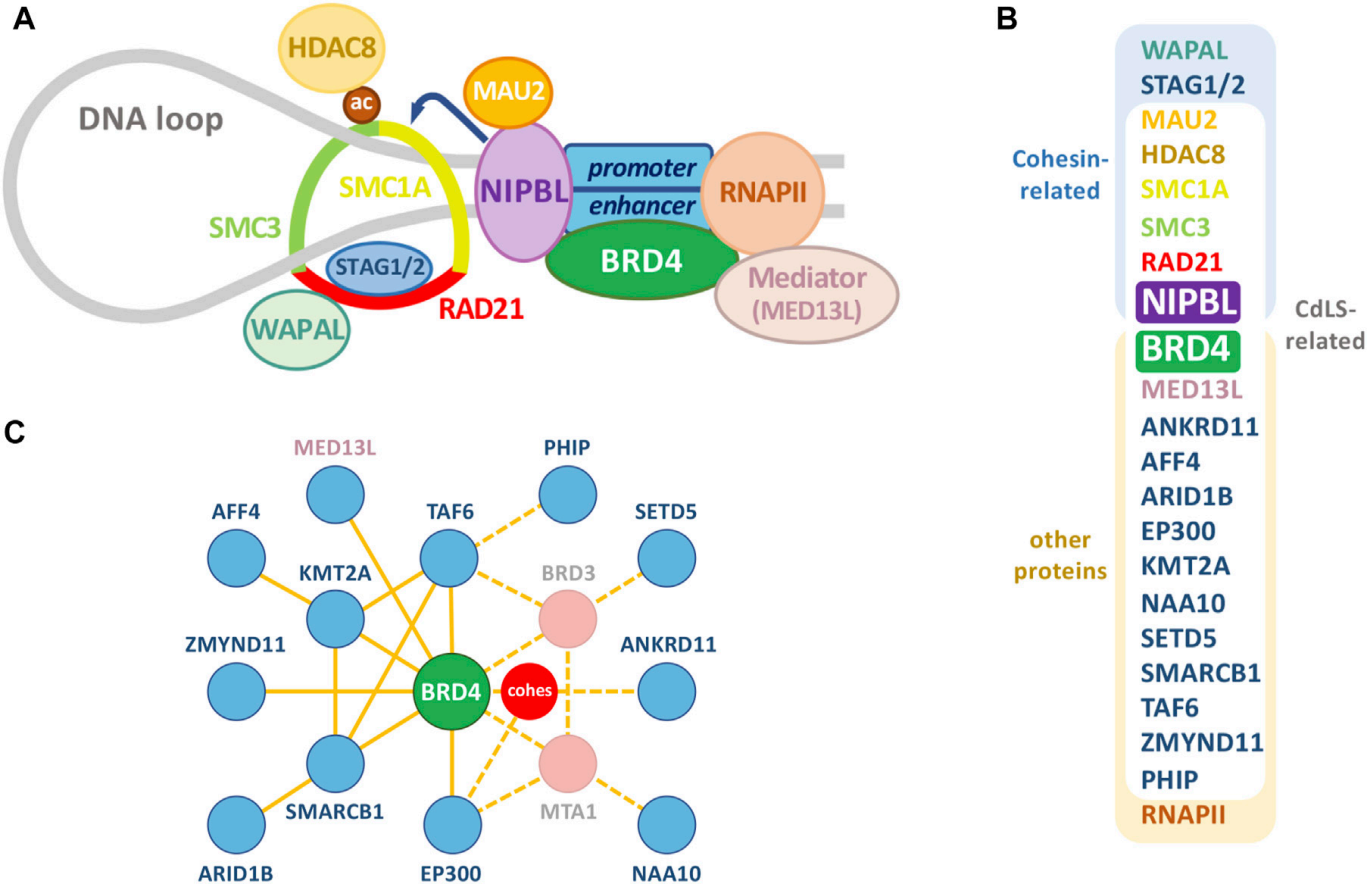
The cohesin complex and associated proteins. **(A)** Relevant proteins of the cohesin complex and other chromatin-associated proteins. Besides RNA polymerase II (RNAPII) and classical CdLS-related proteins, selected NIPBL-BRD4 common interactors, like other cohesin-related proteins and Mediator (see Figure 3), have been depicted. **(B)** Proteins in **(A)**, together with additional factors associated with a CdLS-like phenotype, are classified as cohesin-related and/or CdLS-related. **(C)** Interactome of the additional factors associated with a CdLS-like phenotype (blue) with BRD4 (green) occupying a central position. Dashed lines indicate putative ways of indirect association with BRD4 through the proteins indicated in pink or the cohesin complex (red). Diagram was derived from an esyN tool (http://www.esyn.org/) representation based on BioGRID data.

In the last 2 decades, with the development of genome-wide analysis technologies, CdLS etiology has been associated with mutations in a set of genes functionally related to a chromatin structural or regulatory function. This genetic association has motivated an effort to develop a coherent and exhaustive model of the CdLS phenotypic spectrum reconsidered under the light of the underlying genetic alterations. Nevertheless, this genotypic modelling of CdLS resulted in new layers of complexity, since variants of CdLS-associated genes have been linked to other phenotypes different from CdLS and variants of genes previously associated with other developmental disorders have been found to be linked to cases with features of CdLS (Kline et al., 2018). Besides, genetic variants have been identified in most [over 80% of cases according to an estimation where mosaicism was considered (Huisman et al., 2013)] but not all cases of CdLS so far. Despite this inherent complexity, the first known genetic alterations underlying CdLS were functionally linked to the chromatin-associated cohesin complex, which led to the categorization of CdLS as a genetic cohesinopathy. Nevertheless, not all cohesinopaties result in CdLS (Kline et al., 2018; Piche et al., 2019) and other diseases, as Roberts’ Syndrome (RBS: OMIM 268300), have been also associated to mutations on specific cohesin genes (Ball et al., 2014). In the spectrum of CdLS, the first specific variants identified as probable causative alterations corresponded to the genes *NIPBL*, *SMC1A*, *SMC3*, *RAD21,* and *HDAC8*, all of them directly related with cohesin function (Figures 1A,B). However, more recent screenings have uncovered new genes, in some cases coding for proteins not directly associated with cohesin (See Figure 1B and Table 1) Alterations in different genes have been found in cases with diverse clinical manifestations within the broad CdLS spectrum [see Table 1 for details, and (Kline et al., 2018)] which, together with the frequently observed mosaicism (Huisman et al., 2013), contributes to the complexity of CdLS diagnostic demarcation. In this context, the underlying genetic alterations of the cohesin complex and regulatory factors has provided a useful unifying causative framework for CdLS. In this review, we discuss recent evidence on the function and mechanism of action of CdLS-associated factors that reinforce the notion of transcriptional dysregulation as the main causative alteration underlying CdLS.

**TABLE 1:** Genes associated with CdLS.

Gene	*phen*	*prev*	Other associated developmental syndromes
NIPBL	C	70%	
SMC1A	C/N	5%	Rett syndrome-like Huisman et al. (2017)/Developmental and epileptic encephalopathy 85 (DEE85, OMIM 301044)
SMC3	C/N/A	1–2%	
HDAC8	C/N	4%	
RAD21	N	<1%	Mungan syndrome (OMIM 611376)
ANKRD11	N	<1%	KBG syndrome (KGBS, OMIM 148050)
BRD4	A	<1%	Autosomal dominant syndromic congenital cataracts Jin et al. (2017)
AFF4	A	<1%	CHOPS (OMIM 616368)
ARID1B	A	<1%	Coffin-siris syndrome 1 (CSS1, OMIM 135900)
EP300	A	<1%	Rubinstein-taybi syndrome type 2 (RSTS2, OMIM 613684)/Menke hennekam syndrome 2 (MKHK2, OMIM 618333)
KMT2A	A	<1%	Wiedemann-steiner syndrome (WDSTS, OMIM 605130)
NAA10	A	<1%	Odgen syndrome (OGDNS, OMIM 300855)/Syndromic microphthalmia 1 (MCOPS1, OMIM 309800)
SETD5	A	<1%	Intellectual disability, autosomal dominant 23 (MRD23, OMIM 615761)
SMARCB1	A	<1%	Coffin-siris syndrome 3 (CSS3, OMIM 614608)
TAF6	A	<1%	Alazami-yuan syndrome (ALYUS, OMIM 617126)
ZMYND11	A	<1%	Intellectual disability autosomal dominant 30 (MRD30, OMIM 616083)
PHIP	A	<1%	Chung-jansen syndrome (CHUJANS, OMIM 617991)
MED13L	A	<1%	Intellectual disability and distinctive facial features, with or without cardiac defects (MRFACD, OMIM 616789). Transposition of the great arteries, dextro-looped 1 (D-TGA, OMIM 608808)
MAU2	C	<1%	

*phen.*, phenotype: classic (C), non-classic (N), atypical (A, CdLS-overlapping phenotype), according to (Kline et al., 2018). *prev.*, prevalence. For other references see https://www.omim.org/

### THE Cohesin Complex

Within human cells, thousands of kilobases of DNA have to be organized in a compact yet functional way to fit into the limited space of the nucleus. This complex and dynamic organization is determined to a large degree by structural maintenance of chromosome (SMC) complexes [reviewed in (Sedeño Cacciatore and Rowland, 2019)]. This family of complexes is evolutionarily conserved in procaryotes and eukaryotes, and in eukaryotes includes both condensin and cohesin (Sedeño Cacciatore and Rowland, 2019). Both complexes were originally identified as essential factors for accurate chromosome segregation, involved in mitotic/meiotic chromosome condensation and sister chromatid cohesion, respectively (Strunnikov et al., 1995; Michaelis et al., 1997). Studies conducted over the last decades have shown that besides their classically defined roles in mitosis, both complexes play a role in a number of chromatin-associated processes even though their precise mechanisms of action have not been fully elucidated yet (Wood et al., 2010).

Mitotic cohesin complexes are composed of four core subunits: SMC1A, SMC3, RAD21, and STAG1/2 ((Nasmyth and Haering, 2009), Figure 1A). Besides these core components, many other proteins are required for an efficient cohesin complex function, as the cohesin loading complex NIPBL/MAU2 or the cohesin release factor WAPAL (Figure 1A). The core subunits of the complex can arrange into a ring-shaped heteromeric structure able to topologically entrap DNA molecules (Nasmyth and Haering, 2009) and also to interact with DNA through electrostatic interactions with the Scc3/STAG1 subunit (Li et al., 2018). Thanks to these structural properties, the cohesin complex is able to keep together sister chromatids before they are ready to be conservatively distributed to the daughter cells, which is the classical and nominative function attributed to this complex (Nasmyth and Haering, 2009). However, it was soon clear that besides its mitotic function, the cohesin complex was also required for the proper and functional dynamic chromatin organization during interphase. Several evidences pointed out to this direction, like the loading of cohesin long before its mitotic requirement (Watrin et al., 2006), in a dynamic way (Villa-Hernandez and Bermejo, 2018), enriched at specific chromatin loci (Nasmyth and Haering, 2009) and also present in postmitotic cells (Pauli et al., 2008; Schuldiner et al., 2008; Wendt et al., 2008). A key step in the elucidation of interphasic cohesin function was discovering that, in mammalian cells, most of cohesin binding sites overlap with CTCF (CCCTC-binding factor) binding sites genome-wide (Parelho et al., 2008; Rubio et al., 2008). CTCF is an insulator protein involved in the formation of DNA loops with diverse context-dependent effects on transcription. CTCF does not seem to have a significant effect on global cohesin occupancy on chromatin, but it alters the sites where this complex is retained, probably acting as a boundary factor for the hypothesized DNA loop extrusion mediated by the cohesin complex (reviewed in (van Ruiten and Rowland, 2021)). So cohesin, in partnership with CTCF, is an essential organizer of the interphase genome through the formation of chromatin loops. Also, despite the extensive overlapping of cohesin and CTCF in mammalian cells, a fraction of cohesin binds to chromatin independently of CTCF in differentiated cells, where it contributes to the formation of intrachromosomal loops and to the regulation of the expression of tissue-specific genes in a CTCF-independent fashion (Kagey et al., 2010; Schmidt et al., 2010; Faure et al., 2012).

This capacity of cohesin to form chromatin loops together with its ability to interact with specific chromatin factors places this complex in a central position not only for proper chromatid segregation but for other regulated functions on interphasic chromatin, as DNA damage checkpoint control and repair, DNA replication and transcriptional regulation [reviewed in (Nasmyth and Haering, 2009)]. Given the diversity of nuclear processes that depend on a proper cohesin function, the genetic link between CdLS and the cohesin complex raised the question of which of these functions was more relevant for the pathology of the syndrome. Interestingly, in cells from CdLS patients and in cells derived from mouse models, mitotic chromatid segregation and DNA replication are unperturbed (Tonkin et al., 2004; Kawauchi et al., 2009; Remeseiro et al., 2013). An increased sensitivity to DNA damage due to impaired recombinational repair has been observed in cells from CdLS patients (both with or without detectable NIPBL mutations) subjected to genotoxic stress, but no signs of chromosomal instability could be detected in untreated cells (Vrouwe et al., 2007). Interestingly, in CdLS lymphoblastoid cell lines carrying mutations in either the *SMC1A* or the *SMC3* gene, a downregulation of proteins involved in the response to oxidative stress leads to an increase in global oxidative stress (Gimigliano et al., 2012). In *SMC1A*-mutated CdLS cell lines, this increased oxidative stress has been associated with genomic instability, and it has been shown to be partially rescued by antioxidant drug treatment, raising the question whether this treatment could ameliorate some clinical features recurrent in CdLS patients (Cukrov et al., 2018). However, this phenotype may ultimately rely on gene expression defects (in this case, a downregulation of genes involved in the oxidative stress response). In any case, in all the CdLS models investigated, the cells displayed altered transcription profiles, which provides strong support for the idea that it is a transcriptional deregulation the main cause for the developmental defects in CdLS. In fact, in post-mitotic cells, cohesin has been proven to be continuously required to sustain proper neuronal gene expression (Weiss et al., 2021). However, in the case of cycling cells, since cohesin associates with replication origins (Pherson et al., 2019) and temporal order of DNA replication correlates with chromatin modifications and three-dimensional genome architecture (Klein et al., 2021), it must be better investigated whether the effect on transcription could be related to replication perturbations, at least partially or in certain cellular contexts.

### NIPBL

The first evidence of a cohesin factor regulating developmental gene expression in eumetazoa came from a screen to identify genes that control long-range transcriptional activation of the *Drosophila cut* gene (Rollins et al., 1999). This screen identified the gene *Nipped-B*, named for the gaps in the adult wing margin associated to loss-of-function alleles (Rollins et al., 1999). Nipped-B protein was then postulated to be critical for long-range enhancer-promoter communications (Rollins et al., 1999; Dorsett, 2009), and it was soon linked to the cohesin complex, since the *S. cerevisiae* ortholog Scc2 (together with its partner Scc4) was found to be required for cohesin loading to chromosomes and sister chromatid cohesion (Ciosk et al., 2000; Dorsett, 2009). While the lack of Nipped-B leads to defects in sister chromatid cohesion and premature death, heterozygous *Nipped-B* mutants show only transcriptional effects with no cohesion defects (Rollins et al., 2004).

In humans, the *Nipped-B* homolog, called *NIPBL* [*Nipped-B-*like, (Krantz et al., 2004)], was identified in 2004 as a gene frequently mutated in individuals with CdLS (Krantz et al., 2004; Tonkin et al., 2004). Around 70% of the cases of CdLS investigated so far have been linked to heterozygous mutations in *NIPBL* (Kline et al., 2018), and dozens of different mutations spread along the gene have been identified to date, including frameshift, non-sense, splice-site, and missense mutations (Gillis et al., 2004; Bhuiyan et al., 2006; Yan et al., 2006; Selicorni et al., 2007). The majority of mutations are expected to lead to haploinsufficiency of the protein product, which is now considered the most frequent cause for CdLS, and the one presumably resulting in more severe phenotypes (Kline et al., 2018). This frequency of *NIPBL* mutations underlying CdLS cases could be an underestimation due to undetected mosaicism (Huisman et al., 2013; Ansari et al., 2014), the limited number of samples analyzed and/or to undetected mutations affecting regulatory regions uncovered by current screening strategies (Borck et al., 2006). In fact, two 5′UTR mutations presumably affecting *NIPBL* expression levels have been reported, being both associated to a milder phenotype of CdLS (Borck et al., 2006; Selicorni et al., 2007).

Mutations in other genes have also been reported (reviewed in (Kline et al., 2018), Table 1): mutations in *SMC1A* have been identified in an estimated 5% of individuals with CdLS, usually associated to a non-classical phenotype (Musio et al., 2006; Borck et al., 2007; Deardorff et al., 2007; Ansari et al., 2014; Huisman et al., 2017); a *SMC3* variant has been found in an individual with atypical CdLS (Deardorff et al., 2007) and others have been associated to individuals with some overlapping phenotypical features but not fulfilling the diagnostic criteria of non-classic CdLS (Ansari et al., 2014; Gil-Rodriguez et al., 2015); *RAD21* variants have been found in a small percentage of non-classic CdLS cases ((Deardorff et al., 2012b) and reviewed in (Cheng et al., 2020)); *HDAC8* variants have been also reported, with a typically heterogeneous non-classic phenotype, but also with some classic CdLS cases (Deardorff et al., 2012a; Harakalova et al., 2012; Ansari et al., 2014; Feng et al., 2014; Kaiser et al., 2014; Parenti et al., 2016; Helgeson et al., 2018; Jezela-Stanek et al., 2019); *BRD4* mutations have been associated with few cases of non-classic CdLS (Olley et al., 2018; Alesi et al., 2019); and other gene variants have been identified, mainly by exome sequencing, associated with individuals with limited clinical CdLS features, like variants of *EP300* (Woods et al., 2014; Cucco et al., 2020), *AFF4* (Izumi et al., 2015), *KMT2A* (Yuan et al., 2015; Parenti et al., 2017; Aoi et al., 2019), *NAA10* (Saunier et al., 2016), *TAF6* (Yuan et al., 2015), *ANKRD11* (Ansari et al., 2014; Parenti et al., 2016; Aoi et al., 2019; Cucco et al., 2020), *SETD5, SMARCB1, ARID1B* (Parenti et al., 2017; Aoi et al., 2019), *MAU2* (Parenti et al., 2020), *ZMYND11*, *MED13L* and *PHIP* (Aoi et al., 2019). Since SMC1A, SMC3 and RAD21 are core components of the cohesin complex and NIPBL best known facet was to function as a regulatory component involved in its loading (Dorsett, 2009), it was assumed that genetic variants accounted for an altered cohesin function, which justified the original designation of CdLS as a “cohesinopathy”. Some of the newly identified mutations affect genes coding for proteins that can also be related to cohesin function: HDAC8 mediates the regulatory deacetylation of SMC3 (Deardorff et al., 2012a); BRD4 interacts with NIPBL (Olley et al., 2018; Luna-Peláez et al., 2019) and cohesin (Wong et al., 2014); MAU2 is a partner of NIPBL and EP300 interacts with cohesin and is required for its proper loading after mitosis (Wong et al., 2014). However, for most of the recently identified new variants, no functional relation with cohesin can be established so far, but all of them can be somehow related to a transcriptional function. What is more, given that the majority of cases of CdLS have been linked to *NIPBL* mutations and that individuals with a classic CdLS phenotype are more likely to have variants in *NIPBL* than in any other gene (Kline et al., 2018), together with the fact that *NIPBL* mutation rate may probably be underestimated and that all of the genes involved in CdLS encode proteins somehow linked to transcriptional regulation (many of them with a known link to NIPBL), it seems justified to turn from a classical cohesin-centered paradigm to one more focused in NIPBL (including its pointed role in cohesin functionality together with NIPBL specific functions). Actually, in light of this “NIPBL-centered” model for CdLS, it might be interesting to check if genetic variants other than *NIPBL* indirectly affect NIPBL protein level or functionality. It might also be interesting to check whether in cases with no mutations detected there are subtle reductions in the level of NIPBL due to undetected regulatory alterations that might explain the phenotypical spectrum of CdLS cases according to a dose-dependent effect. In fact, in *Drosophila*, it has been proposed a dose-dependent effect of Nipped-B on gene transcription (Gause et al., 2010).

The prominent role of NIPBL in the etiology of CdLS motivated the research on the function of a so poorly known factor. Since the yeast homolog Scc2 is required for the proper loading of cohesin (Ciosk et al., 2000) and condensin II (D'Ambrosio et al., 2008) complexes, human NIPBL was assumed to function as a cohesin- and condensin II-loading factor. In fact, reductions in the level of NIPBL have been associated with defects in cohesin or condensin II binding to chromatin (Liu and Krantz, 2009; Dowen et al., 2013; Remeseiro et al., 2013; Newkirk et al., 2017), which corroborates the conservation of this functional aspect of NIPBL. More recently, NIPBL role on regulating cohesin function has been expanded to a model with different levels where NIPBL functions not just as a cohesin loader but also by facilitating ATP hydrolysis and loop extrusion (Murayama and Uhlmann, 2014; Haarhuis et al., 2017).

As mentioned above, early research conducted in *Drosophila* models with Nipped-B expanded the functionality of cohesin from mediating sister chromatid cohesion to transcriptional regulation (Rollins et al., 1999; Dorsett, 2009). In this model, it was shown that Nipped-B and cohesin bind preferentially to transcribed regions throughout the entire non-repetitive genome, overlapping with RNA polymerase II (Misulovin et al., 2008). In murine embryonic stem cells (mESC) NIPBL was also found enriched with RNA polymerase II at enhancer and core promoter regions (Kagey et al., 2010), and in human lymphoblastoid cells most binding sites were enriched around promoter regions as well (Zuin et al., 2014b).

Interestingly, in samples from patients and mouse models it has been observed that heterozygous *NIPBL* mutations only reduce the level of *NIPBL* transcript by 30% due to an increased expression from the intact allele through an unknown compensation mechanism (Kawauchi et al., 2009; Liu et al., 2009) and a clinical phenotype is observed with only a 15% reduction in expression (Borck et al., 2006). Although, according to its link to cohesin, it could be speculated about the presence of chromatid-cohesion defects in these samples, with this level of reduction the detected effects on cohesion are mild at best (Kaur et al., 2005; Castronovo et al., 2009; Kawauchi et al., 2009). However, this moderate reduction in the level of NIPBL is sufficient to provoke a global transcriptional dysregulation both in human samples (Liu et al., 2009; Boudaoud et al., 2017; Mills et al., 2018) and mouse models (Kawauchi et al., 2009; Remeseiro et al., 2013; Muto et al., 2014). Interestingly, an effect on DNA repair through homologous recombination has also been appreciated (Vrouwe et al., 2007) and, very recently, alterations in the response to DNA damage were also reported in two lymphoblastoid cell lines derived from CdLS patients with heterozygous *NIPBL* mutations (Olley et al., 2021). However, no remarkable DNA repair defects were detected in *Nipbl* ^*+/-*^ primary mouse embryonic fibroblasts (MEFs) (Remeseiro et al., 2013), so further research would be necessary to precise if this phenotype could be cell-type or NIPBL-level specific and to what extent it represents a global feature and underlying causative factor in the CdLS phenotypic spectrum. Of note, it has been recently described an effect of *Nipbl* heterozygous and *Hdac8* hemizygous mutations in mice on DNA damage, senescence and cytokine secretion in the placenta, suggesting possible negative outcomes for embryonic development (Singh et al., 2020). Even though DNA damage alterations cannot be excluded as a relevant dimension in our comprehension of the complex CdLS syndrome, most studies conducted so far have reach the conclusion that the current evidence suggests that the transcriptional dysregulation is the major etiological substrate for CdLS (Liu and Krantz, 2009). The expression of several genes potentially associated with the phenotypical traits typically observed in CdLS is affected in mouse and human models with NIPBL haploinsufficiency (Kawauchi et al., 2009; Liu et al., 2009; Remeseiro et al., 2013; Muto et al., 2014; Boudaoud et al., 2017; Mills et al., 2018; Luna-Peláez et al., 2019). However, probably due to a limited reduction in the level of NIPBL, in most genes the observed transcriptional effect is moderate, which suggests that phenotypes arise from the collective effects of small changes in the expression of many genes along development (Muto et al., 2011). Also, the differential effect of NIPBL haploinsufficiency on the transcriptome in different cell lineages may contribute to effects, since some genes could be more affected in specific tissues or developmental stages (Kawauchi et al., 2009; Kagey et al., 2010).

In zebrafish models, *nipbl* deficiency has been associated with alterations in the wnt signaling pathway, which seems to account for the increased apoptosis observed in the developing neural tissues (Pistocchi et al., 2013) and with deregulation of the *runx1* gene, which contributes to the observed hematopoietic defects (Mazzola et al., 2020). The cohesin complex is also involved in the regulation of the *runx1* gene but, interestingly, while *nipbl* knockdown reduces *runx1* expression at 30 and 48 h post-fertilization (hpf) (Mazzola et al., 2020), Rad21 (but not CTCF) depletion globally decreases the expression of *runx1* in 13-somites embryos, but increases the relative levels of the P1 transcript at this stage and at 24 hpf (Marsman et al., 2014), suggesting a complex and different regulatory role on this developmental gene. Interestingly, in this model, a dose-dependent effect of Rad21 on transcription of developmental genes has been demonstrated, and this effect takes place within a reduced cohesin background where cell division was not affected (Horsfield et al., 2007), suggesting a cell cycle-independent role for cohesin on transcription (Monnich et al., 2009).

The interaction with cohesin and the evidence in *Drosophila* that Nipped-B mediates long-range transcriptional regulation led to a model where NIPBL would be regulating gene expression by facilitating functional communications between regulatory elements. In fact, it has been pointed out in the *Drosophila* model that Nipped-B-regulated genes with distant regulatory elements, as *cut* and *Ubx*, might be more sensitive to Nipped-B dosage because of combined effects on activation and elongation (Misulovin et al., 2008). In cultured cells from *Drosophila*, Nipped-B and cohesin colocalize and bind preferentially, but not exclusively, to active transcription units (Misulovin et al., 2008). Interestingly, in mESC, NIPBL binds the enhancer and core promoter regions of active genes co-occupied by Mediator [an essential large multisubunit complex involved in the regulation of transcriptional activation, reviewed in (Soutourina, 2018)] and cohesin, but not to those sites co-occupied by cohesin and CTCF, which is consistent with the specific effect of NIPBL reduction on a subset of chromatin-bound cohesin peaks (Kagey et al., 2010; Remeseiro et al., 2013). In some of these loci, with the use of the 3C technology, looping events could be detected between enhancer and promoter regions, and these were dependent on the co-occupation of these factors and the activity of the gene (Kagey et al., 2010). Besides, in zebrafish, three-dimensional fluorescent *in-situ* hybridization revealed that Nipbl and the mediator subunit Med12 are required to bring regions containing long-range enhancers into close proximity within the *hoxda* cluster (Muto et al., 2014). Interestingly, genetic variants of the Mediator subunit MED13L have been associated with CdLS or CdLS-like phenotypes (Aoi et al., 2019). In mESC, NIPBL was found to be enriched at a particular subset of enhancers called super-enhancers (SEs). Initially described in (Hnisz et al., 2013; Whyte et al., 2013), SEs consist of clusters of enhancers that can span as much as 50 kb, densely occupied by transcription factors, transcription complexes (as Mediator) and other chromatin-associated proteins, which are actively transcribed. These SEs are specifically arranged in different cell types, and they associate with the regulation of cell-identity genes (Whyte et al., 2013). In mESC, these SEs are also bound by cohesin and condensin II in a NIPBL-dependent manner, and they regulate ESC-specific genes especially sensitive to perturbations in any of these factors (Dowen et al., 2013). This involvement of NIPBL on gene activation has also been addressed in an inducible system. In a human immortalized neutrophil progenitor cell line (ECOMG), activation with a calcium ionophore rapidly induces the recruitment of NIPBL to thousands of active promoters and enhancers (Zhu et al., 2021). This recruitment occurred with different kinetics: NIPBL accumulated faster and with a greater binding strength at enhancers than at promoters (Zhu et al., 2021). At enhancers, NIPBL occupation was coordinated with an increase in EP300, BRG1 and RNA polymerase II occupancy, while promoter-bound NIPBL is primarily associated with GC-rich DNA sequences (Zhu et al., 2021). Interestingly, in human cells, using a different NIPBL antibody, a sub-population of high-affinity NIPBL binding sites that do not overlap with cohesin and mostly localize at CpG island promoters, could be discriminated from low-enriched NIPBL binding sites co-occupied by cohesin (Zuin et al., 2014b). This observation raised the hypothesis of a possible cohesin-independent function of NIPBL in transcription. In line with a cohesin-independent role of NIPBL, in mouse neural stem cells (NSC) NIPBL was found to be preferentially associated to active and poised promoter and enhancer regions, where it largely co-localizes with the brain-associated transcription factor Zfp609 (van den Berg et al., 2017). On these loci, the overlapping with the cohesin subunit SMC1 was marginal. Instead of an interaction with cohesin, a strong co-localization between NIPBL and the Integrator complex was evidenced. The Integrator complex is a multi-subunit complex conserved in metazoans involved in coordinating the transcriptome and in ensuring proper induction of gene programs [reviewed in (Mendoza-Figueroa et al., 2020)]. Together with Zfp609 and the Integrator complex, NIPBL regulates thousands of genes, many of which are important for neural migration, which underlies the negative effect of NIPBL depletion on neuronal migration, a process speculated to contribute to the cognitive impairment observed in CdLS patients (van den Berg et al., 2017). Interestingly, the NIPBL antibody used for this study was the same used in (Kagey et al., 2010), where they describe significant colocalization of NIPBL and cohesin on promoters and enhancers in mESC, suggesting the existence of cell lineage-specific cohesin-independent functions for NIPBL during development (van den Berg et al., 2017). Also, in agreement with a NIPBL function beyond cohesin, cells from CdLS patients exhibit chromatin decompaction detectable by fluorescence *in situ* hybridization, a phenotype that is not reproduced in normal cells by knocking down the cohesin subunit SMC3 or CTCF (Nolen et al., 2013). This finding also supports the possible role of NIPBL on modulating chromatin architecture, specially affecting genomic regions with highest gene density and highest CTCF and cohesin occupancy, even though this function could be, at least partially, independent of cohesin (Nolen et al., 2013).

With the recent development of genome-wide chromosome conformation capture (Hi-C) maps, new layers of the three-dimensional organization of metazoan chromosomes have been revealed (Dekker et al., 2013). One of these layers corresponds to the so-called TADs (Topologically Associated Domains), regions (that usually span hundreds of kilobases) enriched in contact frequency and with marked boundaries (Dixon et al., 2012). Within these domains, contacts between promoters and regulatory elements are promoted or prevented, which contributes to the structural organization of cell specific transcriptional programs (Dowen et al., 2013). It has been shown that, in mammalian cells, the establishment and maintenance of these self-associating domains require the cohesin complex (Rao et al., 2017; Schwarzer et al., 2017; Rhodes et al., 2020). Depletion of the RAD21 core cohesin component in a human colorectal carcinoma cell line (HCT-116) (Rao et al., 2017) and mESC (Rhodes et al., 2020) eliminates TADs and enhances A/B compartimentalization. And a similar observation has been made after depletion of *Nipbl* in mouse non-dividing hepatocytes (Schwarzer et al., 2017). However, previous experiments of RAD21 depletion in mouse thymocytes (Seitan et al., 2013), mouse post-mitotic astrocytes (Sofueva et al., 2013) and in the HEK293T cell line (Zuin et al., 2014a) have shown a limited impact on chromatin organization at this level. Further investigations on this subject may help to clarify whether the effect of RAD21 or NIPBL depletion on TADs stability might depend on the cell type and/or on the level of the remaining cohesin factor. In fact, it has been suggested an effect of RAD21 depletion on polycomb chromatin domains interactions that is specific to mESC (Rhodes et al., 2020). It has also been argued that this discrepancy about the effect of cohesin depletion on chromatin organization may be due to the fact that low-resolution Hi-C analysis cannot distinguish between loop domains and compartment domains (Rao et al., 2017). It would be also interesting to know if the effect of NIPBL depletion on the genome-wide TAD patterns (Schwarzer et al., 2017) is independent of cohesin to some extent. Unexpectedly, despite the rapid loss of TADs (or “loop domains”) after RAD21 degradation in HCT-116 cells (Rao et al., 2017) and RAD21 depletion in mESC (Rhodes et al., 2020), the effects on global transcription are modest, as they are after RAD21 depletion in the HEK293T cell line (Zuin et al., 2014a). Interestingly, Rao et al. found that, following the degradation of RAD21, a subset of larger loops usually lacking of CTCF remains stable (Rao et al., 2017). The cohesin-independent loops anchors are highly enriched for SEs, where NIPBL has been previously found to be present (Dowen et al., 2013). This observation raises the question of whether NIPBL could be relevant for the organization and/or stability of these cohesin-independent loops. Since perturbations in NIPBL alter cohesin association with chromatin, it is difficult to ascertain to what extent NIPBL could play nuclear roles beyond its regulatory role on cohesin. Of note, in zebrafish, the phenotypes and gene expression changes observed after morpholino knockdown of *nipbl* were different from the ones observed after knocking down the cohesin genes *smc3* and *rad21*, suggesting that functions of Nipbl on gene regulation cannot be ascribed simply to its role in cohesin loading (Muto et al., 2011). Future studies may take this NIPBL functional complexity into consideration by not referring to it just as a cohesin factor, and so it might help to clarify NIPBL range of action.

In a mouse erythroleukemia cell line, NIPBL binds the locus control region (LCR) of the *β-globin* gene and is essentially involved in its activation upon differentiation (Chien et al., 2011). The *β-globin* locus is a classic model to investigate the influence of the communication between distant DNA elements on gene expression, since the interactions between a distal enhancer, the LCR and the *β-globin* genes are required for its proper activation. In a differentiation-induced fashion, NIPBL and cohesin are recruited to regulatory regions and activate *β-globin* genes within the *β-globin* locus, mediating chromatin interactions required for the proper expression of these genes (Chien et al., 2011). Of note, a partial reduction of NIPBL by heterozygous mutation was found to be sufficient to cause alterations in chromatin interactions at the *β-globin* locus, which supports the hypothesis of the alteration of the genome organization and its transcriptional consequences as the main pathomechanism in the etiology of CdLS (Chien et al., 2011). Interestingly, in the *Nipbl* ^*+/-*^ mouse, effects on the expression of genes within the *protocadherin-beta* (*Pcdhb*) cluster depend on the position of the gene in the cluster, being the ones most affected those located at the 3′ and 5′ ends of the cluster [reviewed in (Kawauchi et al., 2016)]. This higher sensitivity of some genes to *Nipbl* inhibition depending on their location in a given chromosomal cluster has also been demonstrated in studies of *hox* genes expression during zebrafish limb development (Muto et al., 2014; Kawauchi et al., 2016).

In lymphoblastoid cells derived from CdLS patients, it has been evidenced a role of NIPBL in the maintenance of the integrity of connected genes communities (Boudaoud et al., 2017). These communities are defined as networks of interacting regions (or nodes) containing at least two genes and noncoding regulatory elements, where the involved genes are usually active and distributed inside a TAD. This concurrency of cotranscribed genes within TADs has been proposed to be at the core of the coordinated regulation of gene networks required to sustain the proper progression of developmental processes [reviewed in (Furlong and Levine, 2018)]. Boudaoud et al. have shown that NIPBL is a component of regulatory regions within connected gene communities, where it tends to occupy a central position and to play a role in the maintenance of the transcriptional integration of the community (Boudaoud et al., 2017). This NIPBL centrality at connected gene communities suggests that *NIPBL* mutations might de-regulate topologically coordinated developmental gene networks in CdLS, contributing to an explanation for the phenotypic spectrum of this syndrome (Boudaoud et al., 2017).

Interestingly, it has been pointed out that the roles of the cohesin complex increase with organismal complexity: while it is involved in chromosome segregation and DNA repair in all eukaryotes, in metazoans, with the emergence of complex developmental programs, it contributes to organize the gene expression networks that sustain and promote this complex development (Dorsett, 2019). The recent discovery that NIPBL interacts with the BET transcriptional coregulator BRD4 (Olley et al., 2018; Luna-Peláez et al., 2019) throws some new light on the complex central role of NIPBL in transcriptional regulation and on the molecular mechanisms underlying CdLS.

### BET Proteins

**FIGURE 2:**
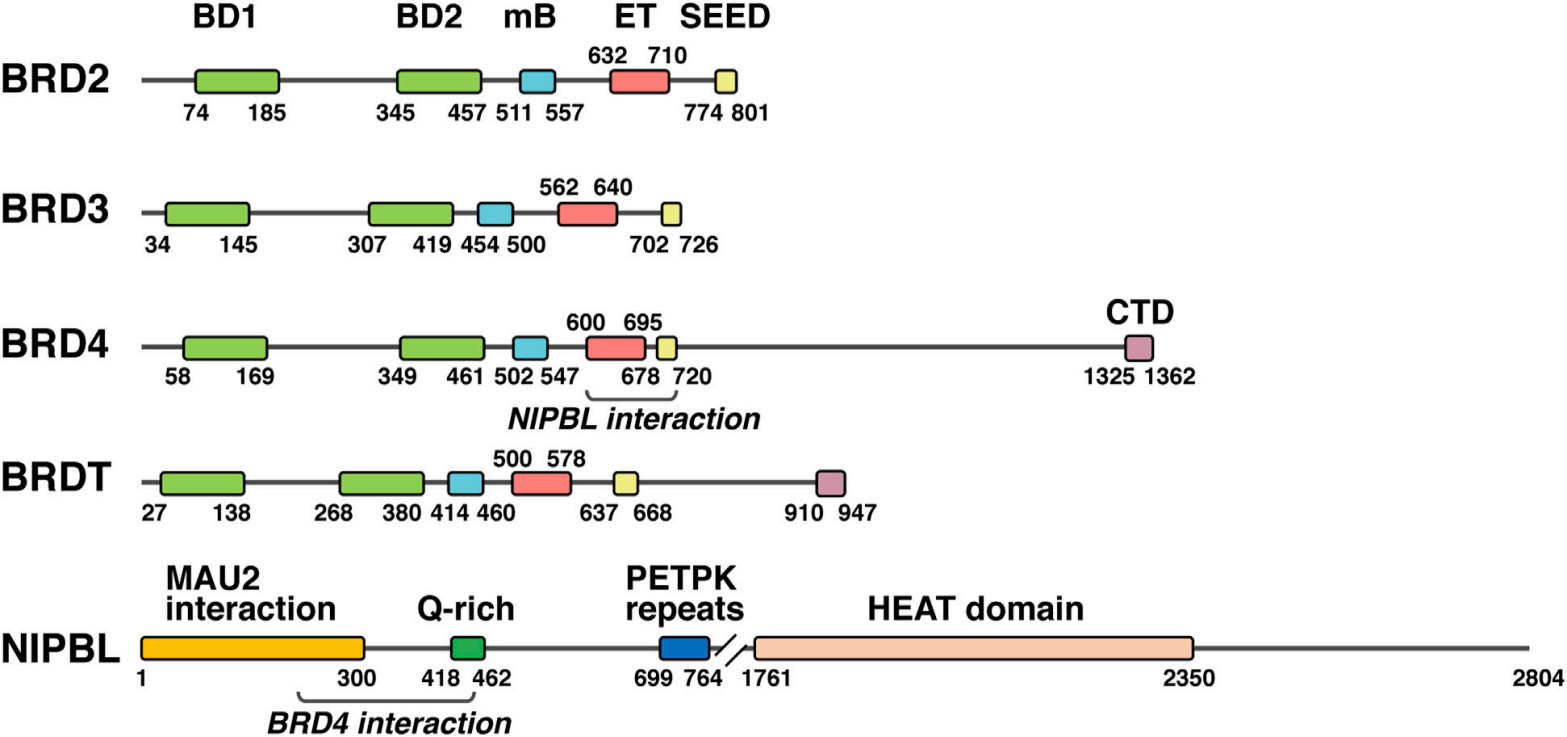
NIPBL and BET proteins. Schematic representation of the different human BET proteins together with NIPBL, indicating the amino acid position (numbers) of main domains and the regions in BRD4 and NIPBL involved in the interaction between them. BD1, bromodomain 1; BD2, bromodomain 2; mB, motif B; ET, extra terminal domain; SEED, SEED domain; CTD, C-terminal domain.

Bromodomain and extra-terminal domain (BET) proteins are chromatin adaptors that act as acetyl-lysine readers on histones and other proteins and have a prominent role in cell cycle control [reviewed in (Taniguchi, 2016)]. Four members, BRD2, BRD3, BRD4, and BRDT are present in vertebrates (Figure 2), and excepting the male germ line-associated member BRDT, rest of proteins are ubiquitously expressed during development and in the adult. Two tandem bromodomains at the N-terminus are involved in acetyl group recognition, while several domains in the C-terminal part account for a variety of functions (Figure 2). The ET domain, as a contact platform for many proteins, is essential for BET function (Rahman et al., 2011; Luna-Peláez et al., 2019), but additional domains N-terminal and C-terminal to the ET domain also account for BET activity. The motif B (mB) contains a coiled coil structure involved in BET dimerization and interaction with other proteins (García-Gutiérrez et al., 2012; García-Gutiérrez et al., 2014; Luna-Peláez and García-Domínguez, 2018), and it has revealed critical for BET function. An additional coiled coil, C-terminal to the ET domain, has also demonstrated to be necessary for BRD2 and BRD3 activity (Werner et al., 2020). In contrast to BRD2 and BRD3, BRD4 and BRDT have a C-terminal extension with a C-terminal domain (CTD) (Figure 2), which has been notably associated with BRD4-mediated control of gene expression (Jang et al., 2005; Yang et al., 2005).

Knock out of *Brd4* in mice leads to embryo death at early postimplantation stages due to the inability of forming the inner cell mass in the blastocyst (Houzelstein et al., 2002). Additionally, reduced cell growth is observed in heterozygous cells either *in vivo* as *in vitro*. Knock out of *Brd2* in mice also leads to embryonic lethality, but at later stages (E11.5–13.5), with associated neural tube abnormalities, as the failure in neural tube closure (Gyuris et al., 2009; Shang et al., 2009). Besides this, altered gene expression, cell growth defects and obesity also associate with lack or reduced levels of BRD2 in mice (Gyuris et al., 2009; Shang et al., 2009; Wang et al., 2009). *Brdt* knock out in mice has evidenced the fundamental role that BRDT plays in spermatogenesis. While mice bearing a truncated BRDT protein lacking the first bromodomain present severe defects in male germ cell differentiation and are sterile, complete knock out leads to meiotic arrest at the end of the prophase (Shang et al., 2007; Gaucher et al., 2012).

Early evidence of the involvement of BET proteins in the maintenance of chromatin organization came from the observation of chromatin decondensation and fragmentation provoked by BRD4 depletion or a dominant negative BRD4 molecule (Wang et al., 2012), and the observation of changes in chromatin organization by ectopic expression of BRDT in non-germ cells (Pivot-Pajot et al., 2003). More recently, the reported cooperation between CTCF and BRD2 for the establishment of architectural and functional chromatin boundaries has illustrated another way of BET participation in preserving chromatin structure (Hsu and Blobel, 2017; Hsu et al., 2017). BET control of chromatin dynamics is exerted, besides direct recognition of acetyl groups on histones, through the interaction with a battery of transcription factors and chromatin complexes. Initial description of BRD2 interaction with E2F was later accompanied by mass spectrometry determination of BRD2 interactors related to core promoter and TATA binding protein-associated factors, Mediator and SWI-SNF complexes and other chromatin/histone modifying enzymes (Denis et al., 2000; Denis et al., 2006). BRD3 interacts with acetylated GATA1 to regulate erythroid genes (Gamsjaeger et al., 2011; Lamonica et al., 2011). An important contribution of BRD4 to transcription regulation is imparted through its interaction with the positive transcription elongation factor b (P-TEFb). Thus, interaction of BRD4 CTD domain with core components of P-TEFb (cyclinT1 and Cdk9) increases phosphorylation of RNA polymerase II C-terminal domain, promoting transcription elongation (Jang et al., 2005; Yang et al., 2005; Itzen et al., 2014). Besides this, it has been demonstrated that a number of factors interacting with the BRD4 ET domain are able to drive transcriptional activation in a P-TEFb-independent manner (Rahman et al., 2011). Wide proteomic studies on ubiquitously expressed BET proteins (Dawson et al., 2011) and more recently on all BET members (Lambert et al., 2019), have significantly increased the list of known BET interactors. Interestingly, mostly BRD2 and BRD4 have been shown also to bind to viral proteins [reviewed in (Weidner-Glunde et al., 2010)], including the recently reported interaction with the SARS-CoV-2 E protein (Gordon et al., 2020).

Wide chromatin localization of at least one BET member at virtually all active genes (LeRoy et al., 2012; Anders et al., 2014) led to the initial assumption of BET involvement in expression of all of them. However, subsequent evidences suggest now specific involvement of BET members in the control of subsets of genes in a cellular context manner [reviewed in (Shi and Vakoc, 2014)]. The different BET members exhibit variable affinities for mono- or di-acetylated histone marks at specific positions, but a major determinant of BET action is through K5 and K12 acetylation on histone H4 (Dey et al., 2003; Kanno et al., 2004; LeRoy et al., 2008; Morinière et al., 2009; Sasaki et al., 2009; Umehara et al., 2010a; Umehara et al., 2010b; Ito et al., 2011). As BET proteins are linked to acetylated chromatin, they are widely considered co-activators, but direct or indirect repressor effects can be also derived from BET activity [see for example (Wang et al., 2009)]. The observation that some BET members remain attached to chromosomes during mitosis (Dey et al., 2000; Dey et al., 2003; García-Gutiérrez et al., 2012) has led to indicate that they are true epigenetic marks, labeling relevant chromatin regions from one generation to the next for transcription regulation (Zhao et al., 2011). Early in the study of BET proteins it became clear their involvement in expression of cell cycle-associated genes, and thereby in cell cycle progression (Denis et al., 2000; Sinha et al., 2005; LeRoy et al., 2008; Mochizuki et al., 2008; Yang et al., 2008). This feature tightly links BET proteins to cancer. Either in classical NUT fusions as independently of NUT, de-regulation of BET expression accounts for many types of cancer [reviewed in (Shorstova et al., 2021)]. This motivated the development of drugs efficiently detaching BET proteins from chromatin as therapeutic molecules to treat cancer (Filippakopoulos et al., 2010; Dawson et al., 2011; Delmore et al., 2011; Mertz et al., 2011; Zuber et al., 2011), but at the same time they proved also to be effective in suppressing inflammation (Nicodeme et al., 2010). To date, the clear therapeutic success of BET drugs is limited to animal models, despite a variety of clinical trials in humans (Bechter and Schöffski, 2020). Drugs are designed on the basis of mimicking the acetyl-lysine group to act as competitors for binding of BET proteins to the chromatin. New strategies like proteolytic targeting chimeras (PROTAC) have recently enriched the therapeutic landscape devoted not only to fight cancer and inflammation but cardiovascular, metabolic, viral and neurological diseases [reviewed in (Andrieu et al., 2018; Singh and Sartor, 2020; Kulikowski et al., 2021)]. Interestingly, BRD2 and BRD4 have been also linked to juvenile myoclonic epilepsy and to learning and memory, respectively (Velisek et al., 2011; Korb et al., 2015).

SEs emerge as essential either for developmental as for physiopathological BET action. BRD4 has been proven to be highly enriched at SEs, and to play a prominent role in their functionality (Chapuy et al., 2013; Lovén et al., 2013). These elements are very relevant in the maintenance of cell identity and in cancer transformation, regulating the expression of oncogenes on which cancer cells become highly dependent (Di Micco et al., 2014; Shi and Vakoc, 2014). An additional term, and potentially closely linked to disease, is latent enhancers, defined as regions in terminally differentiated cells deployed of typical proteins and marks associated with enhancers, but able to acquire them in response to certain stimuli (Ostuni et al., 2013; Kulikowski et al., 2021). They share many characteristics with SEs, and many of them do not return to the basal state once the stimulus ceases, but remain in a memory-mediated latent state for faster and greater induction upon subsequent stimulation. Thus, aberrant activation of latent enhancers by disease-associated noxious stimuli could be efficiently druggable by BET inhibitors.

### NIPBL-BRD4 Interaction

As previously indicated, besides classical cohesin-related factors, a number of additional proteins appear mutated in association with CdLS or CdLS-like phenotypes (Figure 1B; Table 1). As mentioned, the fact that mutations in transcription-related but non-classical cohesin-associated proteins result in CdLS-like phenotypes, strongly suggests that the basis for this developmental syndrome relies on a transcription deficit. We cannot rule out that these transcription regulators are at the end mediating NIPBL recruitment for loading of discrete cohesin amounts at selected chromatin sites, so it is still debated to what extent NIPBL transcription activity can be completely independent of cohesin. For most of the above indicated transcription regulators, a clear physical interaction with cohesin-related proteins has not been established, although ANKRD11, BRD4 and EP300 co-precipitation with core cohesin components has been observed (Wong et al., 2014; Kim et al., 2019). More importantly, it was recently well established that BRD4 associates with NIPBL (Luna-Peláez et al., 2019; Olley et al., 2018). Interestingly, all these CdLS-related factors, directly in most of the cases, are associated with BRD4 (Figure 1C). Thus, non cohesin-related factors associated with a CdLS-like phenotype, might be complexed on regulatory elements to commonly work in related transcriptional tasks. As indicated, a number of these factors have been associated with other syndromes (Table 1), which contributes to the complexity of the CdLS spectrum and its precise diagnosis. Interestingly, this may reflect a true overlapping of certain phenotypic traits underlying mutations in different factors, but converging one way or another in common transcriptional processes during development. In line with this argument, it has been theorized that mutations affecting core regulators of transcriptional machinery associate with phenotypes with overlapping features, as growth problems, facial dysmorphisms and developmental delay/intellectual disability (Izumi, 2016). Examples of these syndromes would be CdLS, Rubinstein-Taybi Syndrome (RSTS; OMIM 180849 and 613684), Coffin-Siris Syndrome (CSS; OMIM 135900, 614608, 614607, 614609, 616938, 615866, 617808, 618027, 618362, 618779, and 618506) and CHOPS Syndrome (OMIM 616368), all of them characterized by global transcriptional dysregulation, and consequently categorized as “disorders of transcriptional regulation” (Izumi, 2016). All of them display similar clinical features, and variants of some genes have been associated to more than one of them (as *EP300* for CdLS and RSTS, *SMARCB1* and *ARID1B* for CdLS and CSS or *AFF4* for CdLS and CHOPS, see Table 1), with only CdLS linked to cohesin function (Izumi, 2016). According to this, instead of being categorized as a cohesinopathy, CdLS could be better considered into a category of disorders of transcriptional regulation, or “chromatin dysregulation disorders” as suggested in (Parenti et al., 2017), together with other syndromes with which it shares not only phenotypical manifestations but also affected genes and nuclear processes (Izumi, 2016). Different mutations in a factor, depending on the altered functional domain, the affected partner association, or on a dose effect, may result in phenotypic differences despite certain overlapping. Re-consideration of CdLS as a transcriptomopathy should not be extended to all cohesinopathies, as other mutations affecting core cohesin components or additional proteins may lead to unambiguous cohesion deficiency. The expanding phenotypes of other cohesinopathies, as well as the underlying genetic alterations, have been recently reviewed elsewhere (Piché et al., 2019).

*BRD4* mutations associated with a CdLS-like phenotype were first reported for individuals with either a deletion encompassing the *BRD4* gene as a *de novo BRD4* missense variant (Olley et al., 2018). Subsequently, a different deletion and a new pathogenic variant have confirmed BRD4 association with CdLS (Alesi et al., 2019; Rentas et al., 2020). Also, in *Drosophila*, genetic interaction between *Nipped-B* and *fs(1)h* (the fly ortholog of the human BRD4 protein) has been reported in the context of cohesin occupancy at enhancers and DNA replication origins (Pherson et al., 2019). The *de novo* mutation reported in (Olley et al., 2018) consisted in a non-conservative amino acid substitution in bromodomain 2 (Tyr430Cys), which leads to impaired binding to acetylated chromatin. This mutation causes a more typical CdLS phenotype than wide deletion encompassing *BRD4*, and interestingly, does not alter the ability to interact with NIPBL. We have explained that this agrees with our identification of the BRD4 ET domain as the contact surface for NIPBL interaction (Luna-Peláez et al., 2019). Moreover, we have reported that both proteins stabilize each other on the chromatin to regulate a common set of developmental genes (Luna-Peláez et al., 2019). Thus, mutations in BRD4 affecting acetyl-group recognition may allow complex formation with NIPBL, but results in the absence of association of the complex with the chromatin. These results suggest that recruitment of complex, and thereby of NIPBL, to the chromatin depends on BRD4, and mechanistically they explain the association of BRD4 mutations with a CdLS phenotype. In fact, there is a significant overlap in the genes differently expressed in both BRD4-and NIPBL-mutant MEFs (Olley et al., 2018). Taking this into consideration, it might be interesting to investigate whether BRD4 mutation (Tyr430Cys) alters NIPBL association with chromatin. Also, the effect of a mild interference with BRD4 association with chromatin, by the use of commercially available specific bromodomain inhibitors, might impair NIPBL binding to chromatin and cause a transcriptional dysregulation phenotype to some degree similar to the one observed upon partial inhibition of NIPBL. A theoretical possibility raised by the BRD4-NIPBL interplay that might be interesting to address is whether increasing levels of BRD4 could compensate to some extent a partial NIPBL deficiency by enhancing its stabilization on the chromatin.

Interestingly, in this mouse model for the BRD4 mutation, despite the transcriptional perturbance observed in MEFs (Olley et al., 2018), the authors did not detect major alterations in the transcriptome in derived mESC (Olley et al., 2021). Instead, they report deficiencies in the regulation of DNA repair in these cells, which highlights the interest of further research on this alteration in additional models of CdLS. This could be also related to the notion that transcriptomic alterations may be cell-type specific during development, which agrees with the different gene-expression alterations detected in different CdLS-model cell types (Kawauchi et al., 2016).

In line with a NIPBL-BRD4 functional interaction, heterozygous mice for both *Brd4* and *Nipbl* genes display some overlapping phenotypes: animals surviving the perinatal period are significantly smaller than wild-type littermates, they display distinctive craniofacial changes in both cases (although in the case of *Brd4* ^*+/−*^ the analysis performed was not as detailed as in the case of *Nipbl* ^*+/-*^ mice) and considerable reduction of subcutaneous body fat and, in both cases, a small yet significant percentage of mice also showed ophthalmological abnormalities (Houzelstein et al., 2002; Kawauchi et al., 2009). Also, brain alterations could be detected in both cases (Houzelstein et al., 2002; Kawauchi et al., 2009), and MEFs derived from *Brd4* ^*+/−*^ mice were highly sensitive to genotoxic agents (Houzelstein et al., 2002), as it was evidenced for CdLS fibroblast and lymphoblast cell lines (Vrouwe et al., 2007).

**FIGURE 3:**
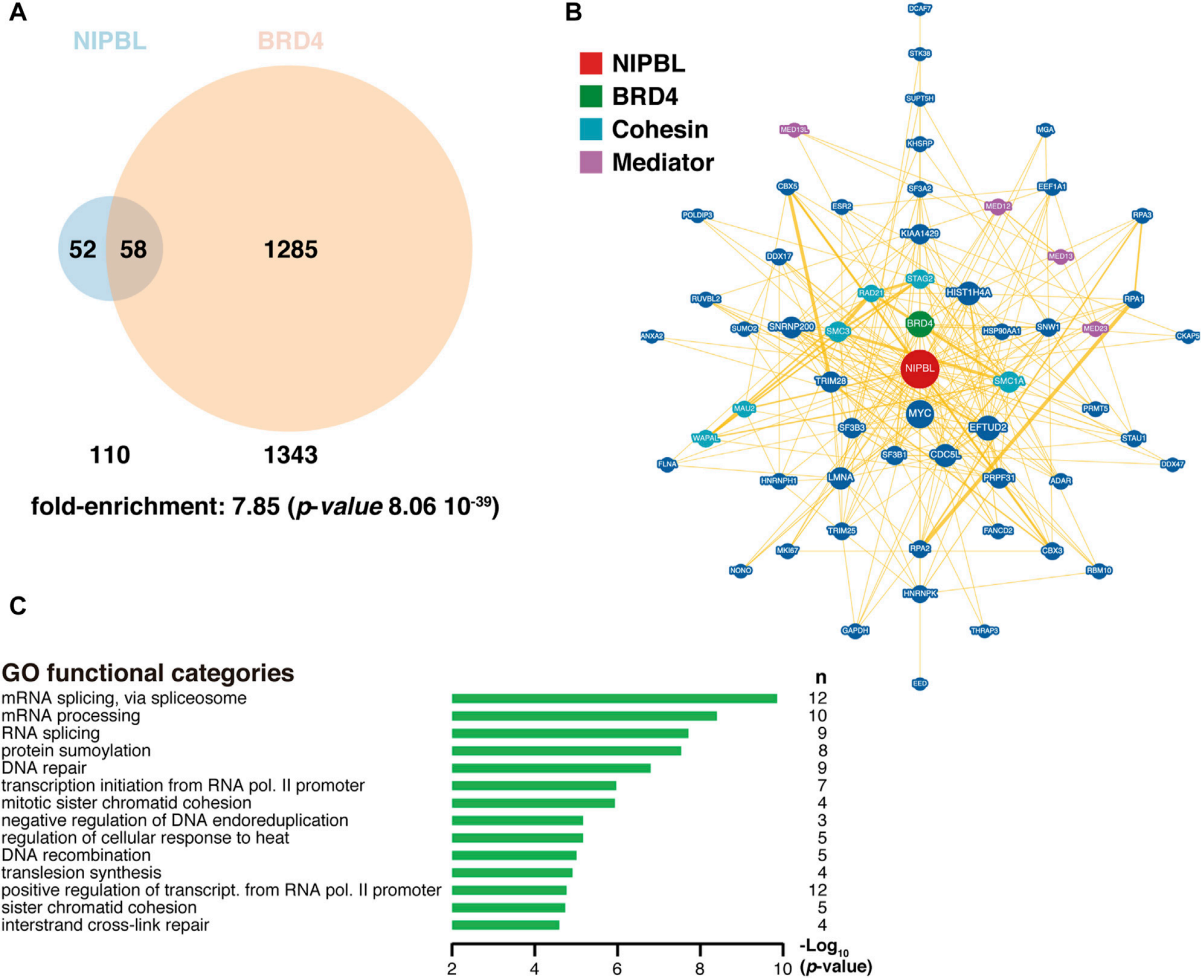
NIPBL and BRD4 interactome. **(A)** Venn diagram indicating the overlap of NIPBL and BRD4 interactors. Fold-enrichment of common interactors and its associated *p*-value from the hypergeometric distribution was obtained at (https://systems.crump.ucla.edu/hypergeometric/index.php), assuming a total proteome of at least 100,000 different proteins. Data were obtained from BioGRID (https://wiki.thebiogrid.org/doku.php/tools). Venn diagrams were constructed with the Venny 2.1 tool (https://bioinfogp.cnb.csic.es/tools/venny/) and depicted with Venn Diagram Maker at (https://www.meta-chart.com/venn). **(B)** Common NIPBL-BRD4 interactome with NIPBL occupying a central position (red). BRD4 is indicated in green, Mediator proteins in purple and cohesin-related proteins in light blue. Data and representation were derived from BioGRID. **(C)** Gene Ontology (GO) analysis of common interactors was performed with the DAVID tool at (https://david.ncifcrf.gov/tools.jsp). n, number of genes in each category.

Also, in agreement with this cooperation, BRD4 and NIPBL share a significant number of interactors (Figure 3A). More than half of the proteins known to interact with NIPBL also interact with BRD4, suggesting that the function of NIPBL could be largely dependent on BRD4 and reinforcing the relevance of their cooperation. In fact, the expression of a truncated version of BRD4 able to bind to the chromatin but lacking the ET domain, leads to dissociation of NIPBL from chromatin (Luna-Peláez et al., 2019). Even though, common interactors with NIPBL represent only a small fraction of all BRD4 known partners, the significance of the overlapping and the fact that NIPBL knockdown also interferes with proper BRD4 binding to chromatin (Luna-Peláez et al., 2019), reflect the important role of NIPBL in BRD4 functionality. The asymmetry in the fraction of co-interactors, besides suggesting NIPBL-independent roles for BRD4, may also reflect the limited knowledge on NIPBL interactome compared to BRD4, a factor for which several proteomic screens have been published uncovering more than a thousand different candidates (Jang et al., 2005; Rahman et al., 2011; Gilan et al., 2016; Zhang et al., 2017; Olley et al., 2018; Mann et al., 2021). Future attempts to complete the NIPBL interactome may change this apparent asymmetry and shed some light on the level of partnership between these two factors. Some of the common interactors identified are related to the cohesin and Mediator complexes (Figure 3B), illustrating a central position of these two factors at the interface of these two essential complexes. Consistently with this, many of the genes coding for these common interactors group to Gene Ontology (GO) categories related to DNA and RNA metabolism (Figure 3C). According to this close partnership between the two factors in the regulation of transcriptional programs in different cellular contexts, as it has been shown for NIPBL in mESC, BRD4 is also bound to SEs in mouse and human ESC, where it is essential for the proper expression of ESC-specific genes and for the maintenance of cell stemness (Di Micco et al., 2014). In human umbilical cord-derived mesenchymal stem cells (MSC), BRD4 inhibition is associated with a downregulation of the WNT pathway (Alghamdi et al., 2016), in line with what was observed for NIPBL (Mazzola et al., 2019). Interestingly, it has been recently reported that cohesin-deficient cells are sensitive to WNT signaling and to BET proteins inhibition (Chin et al., 2020). In light of this NIPBL-BET interplay, it might be of interest to investigate the sensitivity to BET inhibitors in cancer cells with cohesin defects, to check for a higher susceptibility, allowing the use of lower doses of BET inhibitors. In relation to this, sensitivity to treatment with the BET inhibitor JQ1 has been recently shown in two human erythroleukemia cell lines edited to incorporate a previously described homozygous STAG2 mutation (Antony et al., 2020). Both BRD4 and NIPBL are indispensable for the proper hematopoietic development (Dey et al., 2019; Mazzola et al., 2020) and both have been related with hematological defects: it has been detected an increased incidence of thrombocytopenia in CdLS patients (Lambert et al., 2011) and in clinical trials where BRD4 inhibitors have been essayed to treat diverse types of cancer, a prominent highly common toxic effect is thrombocytopenia (reviewed in (Sun et al., 2020)). In all these models where BRD4 and NIPBL functions converge in common regulatory processes, it might be interesting to investigate the effect of interfering with their interaction.

BRD4 is known to stimulate RNA polymerase II promoter-proximal pause release (Jang et al., 2005; Yang et al., 2005; Itzen et al., 2014), and it has been proposed that this function is primarily based on binding to distal enhancers, *via* long-range interactions (Liu et al., 2013; Sancisi et al., 2017). Similarly, NIPBL has been recently proposed to favor productive transcriptional elongation by mediating enhancer-promoter looping (Huang et al., 2020). It would be of interest to confirm whether this newly proposed NIPBL role depends on BRD4.

This recently uncovered interaction between NIPBL and BRD4 contributes to reinforce the notion of CdLS as a disorder based on defects on transcription regulation and to enrich our models on the mechanism underlying NIPBL function, integrating new layers into the functionality of a so complex and central factor for a proper definition of developmental transcriptional programs.

## Concluding Remarks and Future Perspectives

Since its initial association with mutations in cohesin genes, and its consequent categorization as a “cohesinopathy”, there has been an accumulation of evidence in favor of a model of transcriptional dysregulation as the major etiological basis for CdLS. This has led to a reconsideration of the adequacy of this designation, and the term “transcriptomopathy” has been proposed as a better option (Yuan et al., 2015; Kawauchi et al., 2016). Even though the main involved factor in the development of this disorder, NIPBL, has been proven to be critically involved in cohesin functionality, the moderate reduction of its level found in cases of CdLS does not seem to affect chromatid cohesion, while it causes a clear transcriptional perturbance. Despite the effort made in the last decade to more precisely understand the function of this complex factor, it is not clear yet the extent to which it performs transcriptional functions independently of cohesin, as well as its detailed mechanism of action. In this review, we integrate recent findings on CdLS etiological basis and critically re-consider the arguments in favor of a re-conceptualization of CdLS as a transcriptomopathy or a disorder of transcriptional regulation, discussing recent supporting evidence and integrating a new player in the complex NIPBL functional map: its recently identified interacting partner BRD4. Although further studies would be required to better understand the complexity of NIPBL functions on chromatin, the association with BRD4 not only provides new elements to the model, but also opens new possibilities of research. A considerable and solid amount of data have been generated about BRD4 in the last years, a factor that became most interesting after the development of specific inhibitors of its binding to the chromatin that were proven to be successful in preventing the growth of several cancer cellular models. Interestingly, NIPBL has been associated with cancer as well (Barber et al., 2008; Kim et al., 2013; Fazio et al., 2019; Mazzola et al., 2019) and downregulation of NIPBL arrests proliferation of breast cancer cell lines, inducing apoptosis and autophagy (Xu et al., 2015; Zhou et al., 2017), and enhances chemosensitivity of non-small-cell lung cancer cells (Xu et al., 2015; Zheng et al., 2018) and hemangioma stem cells (Liu et al., 2019). So a more detailed knowledge of the functionality of the BRD4 and NIPBL interaction may not only help to better understand the development of CdLS symptomatology but also to expand the therapeutic potential of BRD4 inhibition.
